# Thermal transgenerational effects remain after two generations

**DOI:** 10.1002/ece3.6767

**Published:** 2020-09-13

**Authors:** Who‐Seung Lee, Santiago Salinas, Young‐Rog Lee, Jo Anne Siskidis, Marc Mangel, Stephan B. Munch

**Affiliations:** ^1^ Center for Stock Assessment Research University of California Santa Cruz CA USA; ^2^ NOAA National Marine Fisheries Service Santa Cruz CA USA; ^3^ Environmental Assessment Group Korea Environment Institute Sejong Korea; ^4^ Department of Biology Kalamazoo College Kalamazoo MI USA; ^5^ Department of Biology University of Bergen Bergen Norway

**Keywords:** *Cyprinodon variegatus*, grandparent, growth rate, multigeneration, temperature, transgenerational plasticity

## Abstract

Transgenerational plasticity (TGP) is increasingly recognized as a mechanism by which organisms can respond to environments that change across generations. Although recent empirical and theoretical studies have explored conditions under which TGP is predicted to evolve, it is still unclear whether the effects of the parental environment will remain beyond the offspring generation. Using a small cyprinodontid fish, we explored multigenerational thermal TGP to address two related questions. First (experiment 1), does the strength of TGP decline or accumulate across multiple generations? Second (experiment 2), how does the experience of a temperature novel to both parents and offspring affect the strength of TGP? In the first experiment, we found a significant interaction between F1 and F2 temperatures and juvenile growth, but no effect of egg diameter. The strength of TGP between F0 and F1 generations was similar in both experiments but declined in subsequent generations. Further, experience of a novel temperature accelerated the decline. This pattern, although similar to that found in other species, is certainly not universally observed, suggesting that theoretical and empirical effort is needed to understand the multigenerational dynamics of TGP.

## Graphical abstract

Here, we evaluate the strength of transgenerational plasticity past the first generation in the sheepshead minnow. We show that parental thermal TGP was detected even at the grand‐offspring generation, but that its strength declined across generations.

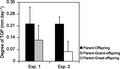

## 1 INTRODUCTION

Changes in climate are happening at an unprecedented pace (Diffenbaugh & Field, 2013; Smith, Edmonds, Hartin, Mundra, & Calvin, 2015), and concomitant increases in temperature are expected to result in changes in metabolism (Dillon, Wang, & Huey, 2010), somatic growth (Urban, Richardson, & Freidenfelds, 2014), population dynamics (Sæther et al., 2000), and ecosystem function (Wrona et al., 2006). These changes are likely to be most dramatic in ectotherms (Deutsch et al., 2008).

Beyond immediate physiological effects, populations can respond to shifts in climate via migration (Pinsky, Worm, Fogarty, Sarmiento, & Levin, 2013), adaptation (Crozier & Hutchings, 2014), or phenotypic plasticity (Charmantier et al., 2008). Phenotypic plasticity *within* generations is well‐studied (Forsman, 2015; Pigliucci, 2001) but a more recent realization is that plasticity can also occur *across* generations (Fox & Mousseau, 1998; Ho & Burggren, 2010; Räsänen & Kruuk, 2007). Such transgenerational effects are expected to evolve when the parental environment provides information on the conditions offspring will face (Simmons, 2011; Via, 1993). Numerous special cases have been studied, including maternal effects, intergenerational environmental effects, anticipatory parental effects, and transgenerational plasticity (TGP). Rather than describe the nuances of each, we narrowly define TGP as a change in reaction norm slope that is driven by the environment in preceding generations. As a consequence, the phenotypic effect of the environment in the parent and offspring generations is nonadditive and cannot be studied in isolation. Given our focus on climate‐driven changes, we are specifically interested in changes in the slope (or shape) of thermal performance curves, rather than changes in elevation.

Formally, we conceptualize TGP as a generalization of the reaction norm approach, defining a mapping between the expected offspring phenotype, *E*(*y*_2_), and the environment in the F1 (parent) and F2 (offspring) generations, *E*_1_ and *E*_2_, respectively, such that $E\left(y_{2}\right)=f\left(E_{1},E_{2}\right)$ and $\left(\partial^{2}f/\partial E_{1}\partial E_{2}\neq 0\right)$. Note that this specification rules out situations in which the environmental effects are purely additive effects such as $E\left(y_{2}\right)=f\left(E_{1}\right)+g\left(E_{2}\right)$. The simplest such model is $E\left(y_{2}\right)=\mu +\alpha_{1}E_{1}+\alpha_{2}E_{2}+\gamma E_{1}E_{2}$. In this case, $\gamma =\partial^{2}f/\partial E_{1}\partial E_{2}$ measures the “interaction” between environments and serves as an index of TGP.

TGP could constitute an important mechanism for coping with climate change by, for example, allowing population persistence until local adaptation occurs (Chevin, Lande, & Mace, 2010; Munday, Warner, Monro, Pandolfi, & Marshall, 2013; Nunney, 2016). Importantly, transgenerational responses to temperature, particularly in ectotherms, are currently neglected in modeling frameworks used to address responses to climate change such as the metabolic theory of ecology (Gillooly, Brown, West, Savage, & Charnov, 2001) and climate envelope modeling (Thomas et al., 2004). Critical to the question of TGP’s value in dealing with rapid and directional changes in climate is how long the phenotypic effects last. In soil mites, the effects of food environment persist across at least three generations (Plaistow, Lapsley, & Benton, 2006) but in Daphnia, TGP in response to a predator cue persists for just two (Walsh, Cooley, Biles, & Munch, 2014). Several other studies have followed epigenetic modulations over multiple generations (e.g., Beemelmanns & Roth, 2017; Dias & Ressler, 2013; Donkin & Barrès, 2018; Gustaffson, Rengefors, & Hansson, 2005; Herman & Sultan, 2011; Zeybel et al., 2012) leading to the conclusion that the duration of a signal can be quite varied (Perez & Lehner, 2019).

Given the relative paucity of studies on the duration of thermal TGP effects in fishes, it is worthwhile to test whether TGP in sheepshead minnows weakens or strengthens after successive generations. To frame our thinking, we extend the model for TGP to grandparental effects, such that the phenotype in offspring (now F3) is given by $E\left(y_{3}\right)=f\left(E_{1},E_{2},E_{3}\right)\approx \mu +\alpha_{1}E_{1}+\alpha_{2}E_{2}+\alpha_{3}E_{3}+\gamma_{12}E_{1}E_{2}+\gamma_{13}E_{1}E_{3}+\gamma_{23}E_{2}E_{3}+\varphi_{123}E_{1}E_{2}E_{3}$. The change in reaction norm due to the grandparent environment is $\partial^{2}f/\partial E_{1}\partial E_{3}=\gamma_{13}+\varphi_{123}E_{2}$.

Specifically, we hypothesize that the growth of F3 individuals will be greatest at the temperatures experienced by F1 individuals, provided that F2 individuals all experienced a common temperature. Based on earlier experiments, we expect this to manifest as a difference in the slope of the growth versus temperature reaction norm in the F3s that is driven by the temperature experienced by their F1 ancestors (i.e., $\gamma_{13}>0$). In addition, since we expect some epigenetic resetting between generations (Kelly, 2014), we hypothesize that—in the absence of a subsequent F2 thermal cue—the F1–F3 interaction effect will be smaller than the F1–F2 interaction (i.e., $\gamma_{13}<\gamma_{23}$).

The relevance of thermal TGP as a mechanism for coping with climate change depends critically on whether the effects accumulate over multiple generations (Burggren, 2015). We therefore tested how the introduction of a novel temperature in the intervening generation modifies the effects of TGP. For species with seasonal reproduction, the breeding season temperature is likely to be positively correlated across years. However, since this correlation typically decays as the number of generations increases, more recent temperatures provide more information about the likely thermal environment for offspring. In light of this, we hypothesize that grandparental (F1) information will be discounted relative to information in the parental (F2) generation, and as a consequence, we predict that a novel intervening temperature will further reduce the magnitude of the F1–F3 interaction (i.e., $\varphi_{123}>0$).

To test these hypotheses, we used sheepshead minnows (*Cyprinodon variegatus*) as our model system. Sheepshead minnows are a small‐bodied (<5 cm), short‐lived (3–4 years) fish found in shallow nearshore waters on the US East Coast from Massachusetts to the Gulf of Mexico. They are tolerant of a wide range of temperatures (−1.5°C–41.6°C; Bennett & Beitinger, 1997), having been caught by us in small ponds that get to 44°C. Sheepshead minnows exhibit thermal TGP (Salinas & Munch, 2012) such that the fastest growing offspring at either 24°C or 34°C were the ones whose parents had experienced the same temperature over 30 days prior to fertilization. This cross‐generation temperature‐matching resulted in 30% faster growth in length relative to offspring of mismatched‐temperature parents.

## 2 MATERIALS AND METHODS

### 2.1 Fish and rearing condition

We caught wild juvenile sheepshead minnows (*Cyprinodon variegatus*) from Florida (FL, 30°20ʹ8″N 87°7ʹ51″W) and South Carolina (SC, 32°45ʹ2″N, 79°53ʹ50″W), USA, in mid‐August in 2010 and 2014, respectively. The wild FL fish were transferred to acclimation aquaria at Stony Brook University, New York, spawned, and their F1 progeny maintained at 22–23°C until the start of experiment 1. The wild SC fish were transferred to the NOAA Fisheries Science Center, Santa Cruz, California, and maintained at 24°C until the start of experiment 2.

Daily care followed standard protocols (Cripe, Hemmer, Goodman, & Vennari, 2009; Salinas & Munch, 2012), including ad libitum feeding of TetraMin flakes (Tetra Holding), 14L:10D photoperiod, and bi‐daily water changes. Salinity was maintained at 20 ppt, but was reduced to 10 ppt for two days prior to egg collection in order to induce spawning in experiment 2.

Among the studies of TGP in fishes, the use of a short‐duration parent temperature treatment as a control for selection on offspring is unique to the experimental design of Salinas and Munch (2012). Unfortunately, this control doubles the size of the experimental design while providing no new information. In the current set of experiments, we have chosen not to repeat the 7‐day exposure to make better use of the available space.

### 2.2 Experiment 1: Persistence of the effects of parental TGP on subsequent generations

Experiment 1 was a continuation of the study by Salinas and Munch (2012). To control for prior temperature history, wild (F0) fish were spawned in the laboratory and the resulting F1 fish were reared to maturity at 21–22°C. At the start of the parental temperature treatment, F1 fish were placed into sea tables (241.3 × 290.2 × 63.5 cm) at each of the experimental temperatures: 24 and 34°C (*n* = 24 females and 18 males in each temperature group). These temperatures represent the range experienced by sheepshead minnows in shallow nearshore habitats in FL (and SC) during the spring and summer. Although 34°C may seem high, sheepshead minnows can tolerate temperatures between −1.5 and 41.6°C (Bennett & Beitinger, 1997).

**FIGURE 1:**
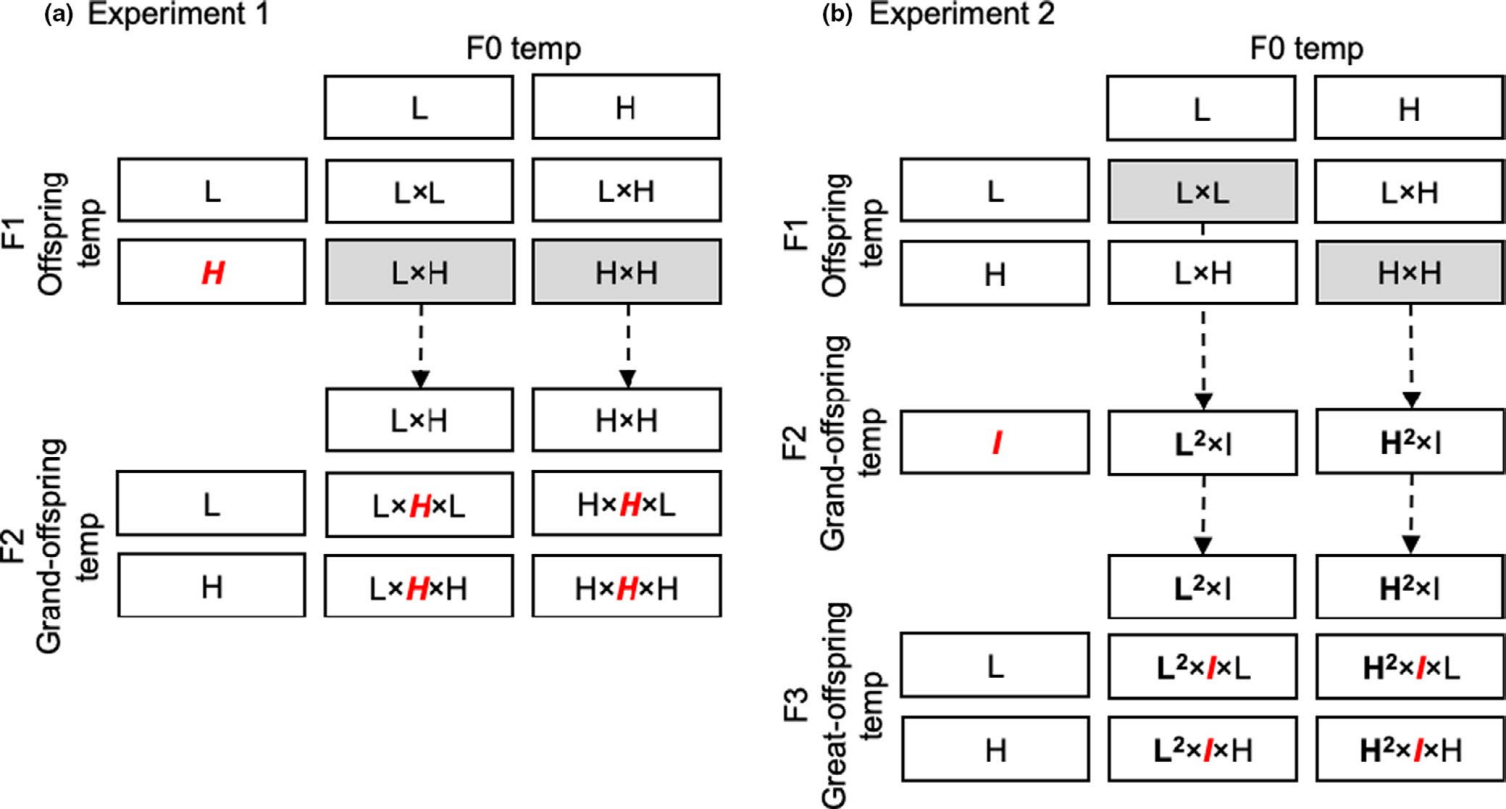
Experimental design in relation to temperature manipulation (L, low; I, intermediate; and H, high)

On the 30th day of the F1 temperature exposure, we collected eggs every 2 hr to ensure that fertilized eggs were exposed to F1 temperatures for as little time as possible. Eggs from each F1 temperature were collected, pooled, and subdivided into batches for rearing at 24 and 34°C (Figure 1). Salinas and Munch (2012) found a significant interaction between F0 (parent) and F1 (offspring) temperatures on F2 growth rate when F1 were exposed to the experimental temperatures for 30 days.

To test for the presence of TGP in the F3 (grand‐offspring) generation, we bred F2 fish from both the (F1→F2) 24→34°C and 34→34°C treatments (henceforth simply 24→34, 34→34) and followed the same egg collection protocol (<2 hr, eggs split into batches and placed at either 24 or 34°C, etc.). We restricted attention to the offspring of F2 fish at 34°C to limit the number of treatment combinations to 4 (Figure 1).

### 2.3 Experiment 2: Effects of a novel temperature in intermediate generation on parental TGP

In mid‐November 2014, we randomly created 64 pairs of male (4.78 ± 0.65 cm) and female (4.55 ± 0.57 cm) wild sheepshead minnows (F0) from SC at 24°C. Each F0 pair was placed in a net‐breeder (26.6 × 16.5 × 16.5 cm) with an egg‐collecting mat and vertical divider to separate male from female in each section. We randomly assigned 32 pairs each to 26°C and 32°C parent temperature treatments. After 30 days of temperature exposure, we removed the vertical divider 30 min before the start of the daily light cycle and collected eggs after 2 hr. All clutches were divided in half among 26°C and 32°C rearing tanks. Upon hatching, we randomly selected four (F1) larvae from each parent pair (some parents had fewer than 4 offspring at a temperature, in which case all offspring were retained). In order to aid identification and to prevent food and social stress, each F1 larva was reared in an individual cylindrical growth chamber (8.5 cm diam × 20.0 cm high) with mesh walls and a solid bottom.

We bred F1 fish from both the (F0→F1) 32→32 and 26→26 treatments. Eggs (F2) were collected, hatched, and reared at 28°C until mature. Again, we used only one temperature treatment in the intervening generation to limit the number of treatment combinations. Here, the F2 temperature differed from both F0 and F1 temperatures in order to test whether a novel temperature (28°C in this study) affects the magnitude of thermal TGP in the subsequent generations.

F2 fish at 28°C from the (F0→F1) 26→26 and 32→32 treatments were spawned, F3 eggs were collected, and clutches subdivided for growth at 26°C and 32°C. Thus, there were four treatment groups: (F0→F1→F2→F3), 26→26→28→26, 26→26→28→32, 32→32→28→26, and 32→32→28→32 (Figure 1).

Thus, this experiment differs from experiment 1 in three ways. First, the 30‐d temperature exposure occurred in F0 rather than F1. Second, the F1 fish were reared at their parent's treatment temperature exclusively. And third, the F2 temperature was intermediate between the treatment (F0, F1) and growth (F3) temperatures. This design is more consistent with a selection experiment, and we are careful to interpret the results accordingly.

### 2.4 Egg diameter and growth rate

In both experiments, eggs were immediately photographed upon collection to measure diameter (±0.001 mm). Approximately every 7 days, we measured standard length from photographs of the fish obtained with a Canon 40D digital camera (3,888 × 2,592 pixels; Canon, Japan) with ImageJ (Schneider, Rasband, & Eliceiri, 2012). Size in juvenile sheepshead minnows is approximately linear through time (Salinas & Munch, 2014). Growth rate was therefore calculated as the difference between length at 6 weeks and length at 2 weeks since spawning divided by time.

### 2.5 Strength of transgenerational plasticity

We calculated the strength of thermal transgenerational plasticity, *d*_TGP_, calculated as

$$d_{\text{TGP}}=\left(G_{L,L}‐G_{H,L}\right)‐\left(G_{L,H}‐G_{H,H}\right)$$

where *L* and *H* are low and high temperatures, respectively, and *G_i,j_* represents the juvenile growth rate of the current generation at *i*°C when the initial generation was held at *j*°C. (e.g., *G*_26,32_ represents growth rate of offspring at 26°C whose grandparents were at 32°C). Note that *d*_TGP_ is equivalent to the interaction term, γ, times the squared difference in temperature between the high and low temperature treatments. So, when the temperature dependence of offspring growth is parallel for all parents, *d*_TGP_ is close to 0 and differs from 0 when there is an interaction between parent and offspring temperatures.

### 2.6 Statistical analysis

Juvenile growth data in all generations, for both experiments, were tested for normality and homogeneity of variance; we then analyzed these data using a two‐way ANCOVA treating temperature of all generations as fixed effects and using egg diameter as a covariate. Analysis in experiment 2 further included family membership as a random effect. We used chi‐square goodness‐of‐fit tests (Sokal & Rohlf, 1995) to evaluate whether the effect of parental TGP remained after the first generation. We used power analysis for ANCOVA to assess the robustness of the results using G*Power (Faul, Erdfelder, Lang, & Buchner, 2007) with power calculated as 1‐(Type II error). All statistical analyses were performed using R 3.3.0 (R Development Core Team, 2016).

## 3 RESULTS

**FIGURE 2:**
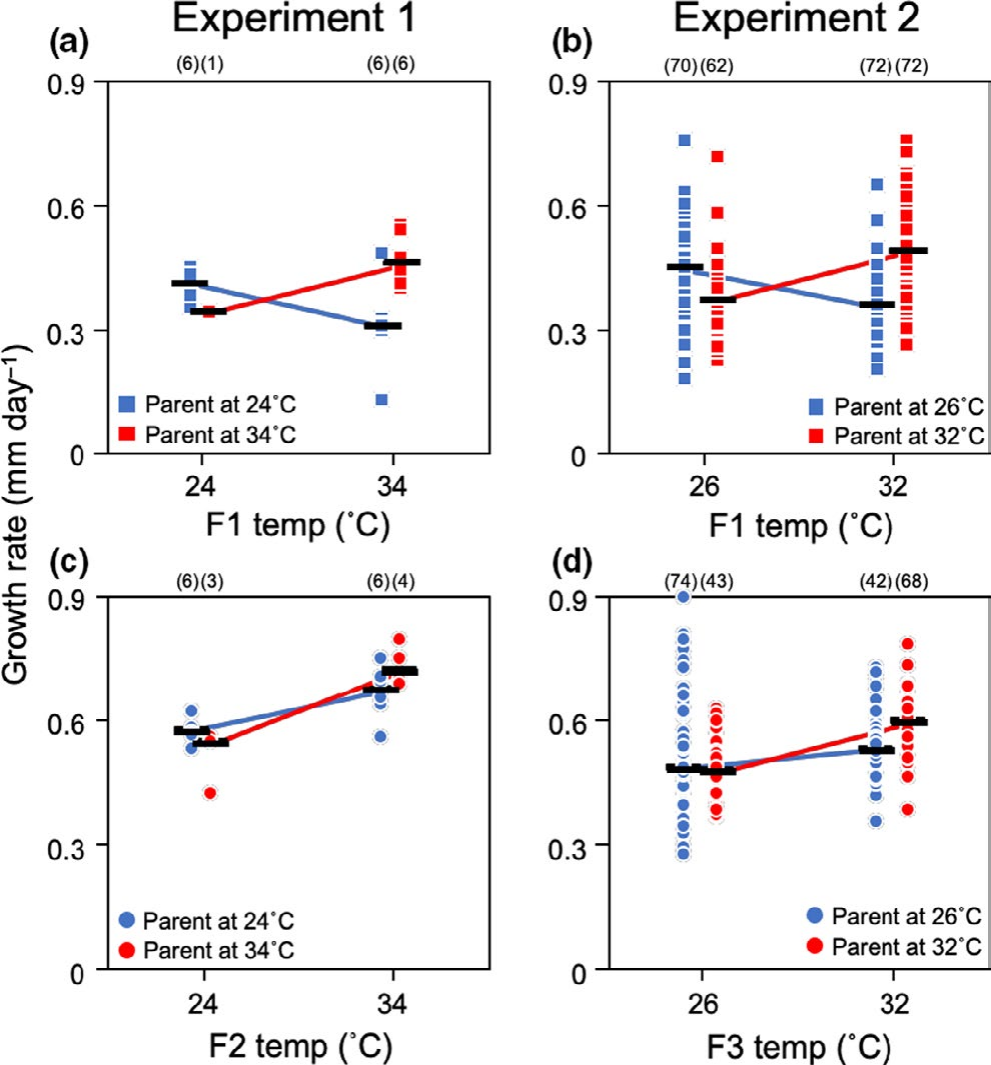
Experiment 1 (left side)—growth rate of (a) F1 and (c) F2 at 24 and 34°C from parents held for 30 days at 24 (blue symbols) and 34°C (red symbols). Experiment 2 (right side)—growth rate of (b) F1 and (d) F3 at 26 and 32°C from parents (F0 and F1 in experiments 1 and 2, respectively) held for 30 days at 26 (blue symbols) and 32°C (red symbols). The black lines mean the median of each group. In both experiments, effects of the interactions between parent and following generations’ temperature on juvenile growth are significant (*p* < .001, see Tables 1 and 2). Sample sizes (number of temperature treatment group in experiment 1; number of individuals in experiment 2) are shown in parentheses

In experiment 1, we found a significant effect of the interaction between F1 and F2 temperatures on juvenile growth in F1 (Table 1, Figure 2a), with matched offspring (same temperature across generations) outperforming mismatched ones. F1 temperature affected juvenile growth in F2, but there was no effect of F2 temperature (power = 0.933). In addition, we found no effect of egg diameter on juvenile growth on F2 (power = 0.957). Similarly, juvenile growth in F3 was significantly affected by the interaction between F1 and F3 temperatures (Table 1, Figure 2c), again with matched individuals growing faster. F3 temperature significantly affected juvenile growth on F3, whereas there were no effects of F1 temperature (power = 0.677) or egg diameter (power = 0.727) on F3 growth (Table 1).

**TABLE 1:** Two‐way ANCOVA results with F0, F1, and F2 temperature as factors and egg diameter as covariate for experiment 1

Source	*df*	MS	*F*	*p*
F0 and F1				
Egg diameter (mm)	1	0.010	1.957	.184
F0 temp (°C)	1	0.039	8.025	.013
F1 temp (°C)	1	0.009	1.772	.204
F0 temp × F1 temp	1	0.045	9.266	.009
Error	14	0.005		
Total	18			
F0 and F2				
Egg diameter (mm)	1	0.001	0.050	.826
F0 temp (°C)	1	0.001	0.210	.654
F2 temp (°C)	1	0.088	25.371	<.001
F0 temp × F2 temp	1	0.021	5.992	.028
Error	14	0.003		
Total	18			

In experiment 2, we found that juvenile growth in F1 was significantly affected by the interaction between F0 and F1 temperatures (faster growth in matched treatments; Table 2, Figure 2b). While there was no direct effect of F1 temperature on juvenile growth (power = 0.987), F0 temperature did affect juvenile growth in F1 (Table 2). There was no effect of egg diameter on F1 growth (power = 0.978, Table 2). Growth in F3 was similarly affected by the interaction between F0 and F1 temperatures (Table 2, Figure 2d). F3 temperature also affected F3 growth, while there were no direct effects of F0 temperature (power = 0.890) and egg diameter (power = 0.983, Table 2).

**TABLE 2:** Two‐way ANCOVA results with F0, F1, and F3 temperature as factors and egg diameter as covariate for experiment 2

Source	*df*	MS	*F*	*p*
F0 and F1				
Egg diameter (mm)	1	0.010	0.790	.375
F0 temp (°C)	1	0.124	9.355	.003
F1 temp (°C)	1	0.001	0.069	.793
F0 temp × F1 temp	1	0.577	43.639	<.001
Error	210	0.013		
Total	214			
F0 and F3				
Egg diameter (mm)	1	0.024	2.799	.095
F0 temp (°C)	1	0.015	1.779	.183
F3 temp (°C)	1	0.291	33.576	<.001
F0 temp × F3 temp	1	0.060	6.868	.009
Error	271	0.009		
Total	275			

**FIGURE 3:**
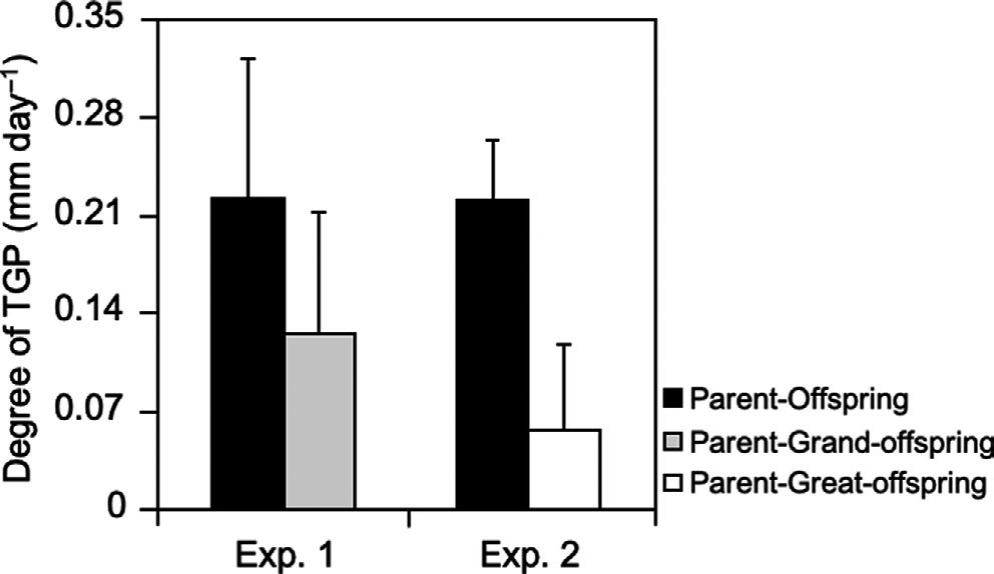
Strength of TGP, quantified as the interaction of juvenile growth in offspring, grand‐offspring or great‐offspring and parent temperatures (see Section 2). Note that temperatures in parents, offspring, and grand‐offspring were 24 and 34°C (experiment 1) and temperatures in parents, offspring, and great‐offspring were 26 and 32°C (experiment 2)

The degree of TGP, d_TGP_, between parents and offspring was similar in experiments 1 and 2, 0.222 ± 0.100 and 0.220 ± 0.044, respectively (see Figure 3). In both experiments, the strength of TGP declined by the F3 generation (expt 1:0.126 ± 0.087 and expt. 2:0.057 ± 0.060) (Figure 3). In addition, the strength of TGP between F0 and F3 in experiment 2 was about 50% less than the strength of TGP between F1 and F3 in experiment 1 (chi‐square goodness‐of‐fit, *χ* ^2^ = 5.521, *df* = 1, *p* = .019; Figure 3). In summary, as we move from 1 to 2 to 3 generations removed, the strength of the transgenerational effect goes from 0.22 to 0.12 to 0.06, decreasing approximately by half with each step.

## 4 DISCUSSION

Our results indicate that parental thermal TGP persisted in subsequent generations (F2 in experiment 1 and F3 in experiment 2), but that its strength declined across generations. Furthermore, when the intermediate (F2 in experiment 2) generation experienced a novel temperature, the strength of TGP in the subsequent (F3) generation was reduced. We found no effect of egg diameter, which suggests that the decline of TGP across generations was not related to maternal provisioning.

Although these results are consistent with our hypotheses for transgenerational effects, we must concede the possibility that selection plays a role in these results as there was non‐negligible mortality in the F2 generation in experiment 2 (4.2%). Nevertheless, the results are consistent with previous theoretical (e.g., Prizak, Ezard, & Hoyle, 2014) and experimental (e.g., Beemelmanns & Roth, 2017; Hafer, Ebil, Uller, & Pike, 2011) work on TGP. For example, Walsh et al. (2014) exposed individual clones of Daphnia to different environmental treatments and then followed the response over several generations. Daphnia reared in the same environment as their mother exhibited greater transgenerational responses than offspring in mismatched environments. As in the minnows, the transgenerational response in Daphnia decreased from F2 to F3 by roughly 50% and was no longer evident by F4. Since these changes in phenotype are observed *within* clones, they cannot possibly be the result of selection. Rather, they are most parsimoniously explained by some epigenetic mechanism. Indeed, Schield et al. (2016) subsequently showed that significant changes to methylation patterns coincide with these phenotypic changes across generations.

When parents receive cues about the probable offspring temperature (Mousseau & Fox, 1998), we expect offspring to grow faster when parents predict correctly (i.e., when there is a high correlation between parental cue and offspring environment). In both experiments, we found faster growth when temperatures between generations were matched (Figure 2). In addition, the response to ancestral temperatures declined with the number of intervening generations, which is consistent with the expected decline in correlation between temperatures across multiple years.

This pattern is not universal, however (see examples in Bell & Hellmann, 2019). Additionally, there are many other features of multigeneration TGP dynamics worthy of further exploration. For instance, the effect of guppy (*Poecilia reticulata*) grandparents’ temperature differently impacted swimming performance in males and females (Le Roy, Loughland, & Seebacher, 2017). In stickleback (*Gasterosteus aculeatus*), F1 mothers at a high temperature produced smaller F2 offspring at high temperature but relatively larger ones in the F3 generation (Shama & Wegner, 2014). Further, how the environmental variable changes between generations can also modify TGP strength (Donelson, Wong, Booth, & Munday, 2016).

The type of inheritance system, the reliability of the cue, the effectiveness of the sensory mechanism, and the fidelity of the information transfer all have important consequences for the evolution of growth in thermally changing environments (Badyaev & Uller, 2009; Shea, Pen, & Uller, 2011). Based on our and others’ results, it is increasingly clear that we need modeling efforts aimed at integrating various streams of information, including genetic, developmental, parental, and grandparental effects (Day & Bonduriansky, 2011; Leimar & McNamara, 2015) for accurate predictions of population changes in response to environmental perturbations.

## CONFLICT OF INTERESTS

None declared.

## AUTHOR CONTRIBUTION

**Who‐Seung Lee:** Conceptualization (equal); Formal analysis (lead); Investigation (equal); Methodology (equal); Writing‐original draft (lead); Writing‐review & editing (equal). **Santiago Salinas:** Conceptualization (equal); Investigation (equal); Methodology (equal); Writing‐original draft (lead); Writing‐review & editing (equal). **Young‐Rog Lee:** Investigation (equal). **Jo Anne Siskidis:** Investigation (equal). **Marc Mangel:** Conceptualization (equal); Funding acquisition (equal); Methodology (equal); Supervision (equal); Writing‐review & editing (equal). **Stephan B. Munch:** Conceptualization (equal); Funding acquisition (equal); Methodology (equal); Project administration (equal); Supervision (equal); Writing‐review & editing (equal).

## Data Availability

Data are deposited on Dryad: https://doi.org/10.5061/dryad.s1rn8pk5j.
